# High–Energy–Density Fiber Supercapacitor Based on Graphene-Enhanced Hierarchically Nanostructured Conductive Polymer Composite Electrodes

**DOI:** 10.3390/nano15171350

**Published:** 2025-09-02

**Authors:** Chuangen Ye, Qingfeng Yang, Mingxian Xu, Haitang Qiu, Xiaozhen Zhang, Jianping Ma, Haiyang Gao, Xuansheng Feng, Yong Li

**Affiliations:** 1 High-tech Industry (Pilot) Base, Advanced Materials Institute, Qilu University of Technology (Shandong Academy of Sciences), Jinan 250014, China; 2 Qingdao Campus of Naval Aviation University, Qingdao 310018, China; 3 Shandong Sacred Sun Power Sources Co. Ltd., Qufu 273100, China

**Keywords:** fiber-shaped supercapacitors, CNT, graphene, polyaniline

## Abstract

The development of portable and wearable electronics has promoted the advancement of fiber supercapacitors (FSCs), but their low energy density still limits their application in flexible devices. Herein, we incorporated micron-sized graphene dispersions at varying concentrations into the polyaniline (PANI) precursor solution prepared via electrochemical polymerization and subsequently electrodeposited PANI/graphene composites onto the surface of carbon nanotube (CNT) fibers, ultimately obtaining fibrous PANI/graphene@CNT composite electrodes. This electrode material not only exhibits the superior electrochemical activity characteristic of conducting polymers synthesized by electrochemical polymerization but also possesses a relatively high specific surface area. Furthermore, we fabricated coaxial fiber supercapacitors using PANI/graphene@CNT composite fibers and CNT films as the positive and negative electrode materials, respectively. The maximum energy density and power density could reach 160.5 µWh cm^−2^ and 13 mW cm^−2^ respectively, proving its excellent energy storage and output capabilities. More importantly, the prepared CFASC device showed remarkable mechanical and electrochemical durability. Even after 3000 bending cycles, it retained 89.77% of its original capacitance, highlighting its promising applicability in the realm of flexible electronics. The resulting devices demonstrate excellent electrochemical performance and mechanical stability.

## 1. Introduction

In recent years, driven by the rapid advancement of wearable technology, energy storage devices that power these systems have also evolved to become more flexible, lightweight, and efficient. Among various flexible power sources, fiber-shaped supercapacitors (FSCs) have garnered increasing attention due to their advantageous properties, including flexibility, weavability, excellent safety, high power density, and rapid charge–discharge capabilities [1,2,3]. Nevertheless, the development of FSCs remains in its early stages, with several challenges yet to be addressed. The primary challenge lies in enhancing the energy density of FSCs without compromising other performance metrics [4,5].

The progress in flexible FSCs is closely tied to ongoing advancements in carbon material research. Most FSCs are constructed using carbon-based fibers, which offer excellent mechanical strength, high electrical conductivity, and a large specific surface area. FSCs fabricated exclusively from carbon materials operate solely in the electric double-layer capacitance (EDLC) mode, which inherently limits their energy density [6]. To overcome this limitation and enhance energy density, researchers frequently employ carbon-based fibers as substrates and improve electrode electrochemical performance by depositing active pseudocapacitive materials onto their surfaces [7,8]. Commonly used pseudocapacitive materials include transition metal oxides and conductive polymers [9,10,11]. Compared with transition metal oxides, conductive polymers offer several advantages for the development of flexible supercapacitors: (1) conductive polymers are lightweight and flexible, meeting the material requirements for flexibility, while their polymer chain structure enhances the mechanical properties of composite electrodes; (2) they exhibit excellent electrical conductivity. In contrast, the poor conductivity of transition metal oxides has long been a drawback, as it increases internal resistance and negatively affects both power density and cycling stability; (3) conductive polymers are cost-effective and do not require the use of scarce or precious metals [12,13,14,15]. However, their practical application is limited by relatively low conductivity. As a result, specific capacitances of 150–190 F g^−1^ for polyaniline (PANI), 80–100 F g^−1^ for polypyrrole (PPy), and 78–117 F g^−1^ for polythiophene (PTh) have been reported in both aqueous and non-aqueous electrolytes, respectively [16,17]. These values are significantly lower than theoretical expectations and may not satisfy practical requirements [18]. Incorporating conducting polymers with carbon materials appears to be an effective strategy, as it can not only significantly enhance the specific capacitance of carbon materials but also further improve the specific capacity of conducting polymers by enhancing their conductivity [19,20,21].

Nanocomposites composed of conductive polymers and carbon materials are typically synthesized through chemical or electrochemical polymerization of monomers [22]. Although the electrochemical method yields more homogeneous deposits with enhanced electrochemical activity, the chemical method is preferred due to its cost-effectiveness and the ease with which porous composites can be produced [23]. Moreover, the porosity of chemically synthesized composites is significantly higher compared to the denser structures formed electrochemically [24]. Porous structures facilitate the rapid penetration of the electrolyte into the active polymer, thereby creating optimal conditions for efficient ion diffusion and migration within the polymer matrix, which significantly enhances electrode performance [25,26]. Therefore, developing an electrochemical polymerization strategy that enables the formation of CP/carbon composites with both excellent electrochemical activity and high porosity is crucial for the more effective utilization of conductive polymer materials. There is strong evidence that such a nanotextured composite structure is ideal for rapid ionic diffusion and migration within the polymer matrix, thereby significantly enhancing electrode performance [27,28]. Similarly, graphenes, which also possess a mesoporous and flexible nature, are expected to yield comparable beneficial effects [29].

Herein, micron-sized graphene dispersions with varying concentrations were incorporated into the polyaniline (PANI) precursor solution prepared via electrochemical polymerization. Subsequently, PANI/graphene composites were electrodeposited onto the surface of carbon nanotube (CNT) fibers through the same electrochemical polymerization process, resulting in fibrous PANI/graphene@CNT composite electrodes. This electrode material not only retains the superior electrochemical activity characteristic of electrochemically polymerized conductive polymers but also exhibits a relatively high specific surface area. The presence of pore structures across different size scales enhances the contact area between the electrolyte and the active materials while simultaneously facilitating rapid ion diffusion and migration within the polymer matrix. Furthermore, coaxial fibrous supercapacitors were assembled using PANI/graphene@CNT composite fibers and CNT films as the positive and negative electrode materials, respectively. The resulting devices demonstrate outstanding electrochemical performance as well as excellent mechanical stability.

## 2. Materials and Methods

### 2.1. Fabrication of PANI@CNT and PANI/Graphene@CNT Fiber Electrodes

Graphene dispersion (99wt%, 2 mg mL^−1^), carbon nanotube (CNT) fiber, and CNT film were obtained from Suzhou Tanfeng Graphene Science and Technology Co., Ltd., Suzhou, China. The graphene dispersion is obtained by the stable dispersion of graphene prepared by Hummers method after being functionalized [30]. The graphene dispersion exhibits a purity of 98%, with a monolayer content exceeding 90%, and the average sheet diameter ranges from 0.2 to 10 μm. The CNT film was produced through an enhanced chemical vapor deposition technique, as described in prior studies [31]. The CNT fiber was fabricated by in situ shrinking of a CVD—grown CNT film using water.

The in situ electro-polymerization method was employed to deposit polyaniline (PANI) and PANI/graphene shells onto the surface of CNT fibers, respectively, to obtain PANI@CNT and PANI/graphene@CNT composite electrodes. Initially, an aqueous solution containing 0.5 M aniline and 1 M sulfuric acid was prepared as the precursor solution for the PANI@CNT composite electrode. The preparation procedure was as follows: 3.73 g of aniline monomer was added dropwise to 80 mL of a 1 M sulfuric acid aqueous solution, followed by vigorous magnetic stirring for more than 20 min until a clear and transparent solution was obtained. The precursor solution for preparing the PANI/graphene@CNT composite electrode was prepared by adding different amounts of graphene dispersion to this solution, with the addition amounts of graphene dispersion being 0.1 mL, 0.2 mL, 0.3 mL, 0.4 mL, 0.5 mL, and 0.6 mL, respectively.

In situ electrochemical polymerization was conducted within a three-electrode system, utilizing CNT hydrogel fiber, a Pt electrode, and an Ag/AgCl electrode as the working electrode, counter electrode, and reference electrode, respectively. The precursor solution prepared above served as the electrolyte. The electrochemical parameters were set as follows: scanning voltage range of 0–0.8 V, scanning rate of 100 mV s^−1^, and 200 cycles. Subsequently, the obtained PANI@CNT and PANI/graphene@CNT composite electrodes were rinsed several times with deionized H_2_O and then dried at 70 °C for 1 day.

### 2.2. Assembling of the CFASC

The H_2_SO_4_-PVA gel electrolyte was synthesized through the following procedure: 10 g of H_2_SO_4_ was mixed with 100 mL of deionized water, followed by the addition of 10 g of PVA powder. The resulting mixture was gradually heated to approximately 90 °C under vigorous magnetic stirring until a clear solution was obtained. The prepared PANI/graphene@CNT composite electrode was immersed in the H_2_SO_4_-PVA gel electrolyte for 30 s and subsequently transferred to ambient air to facilitate complete solidification of the gel electrolyte. A CNT film was cut into 3 mm-wide strips, which were then immersed in the H_2_SO_4_-PVA gel electrolyte and spirally wound around the PANI/graphene@CNT composite electrode. Following full solidification of the electrolyte, a coaxial fiber-shaped asymmetric supercapacitor (CFASC) was successfully fabricated. The preparation process and structural configuration of the PANI/graphene@CNT//CNT-film CFASC device are schematically illustrated in Figure 2b,c.

### 2.3. Materials Characterization and Electrochemical Measurements

The microstructure of the electrode materials was analyzed using a field emission scanning electron microscope (FESEM, Hitachi S-4800, Osaka, Japan). Raman spectroscopy was performed with a LabRam-1B (HORIBA, Ltd., Lyon, France) spectrometer employing a He-Ne laser at an excitation wavelength of 532 nm. The phase component of the samples was investigated by X-ray diffraction (XRD, Smartlab, Rigaku Corporation, Tokyo, Japan) with Cu Kα radiation (λ = 1.540 Å). The bonding characteristics were obtained from X-ray photoelectron spectroscopy (XPS, ESCALAB 250, ThermoFisher Scientific, Waltham, MA, USA). An electrochemical workstation (CHI 660E, CH Instruments, Inc., Shanghai, China) was used to evaluate the electrochemical performance of the electrodes and CFASC devices. The main test methods are cyclic voltammetry (CV), electrochemical impedance spectroscopy (EIS), and galvanostatic charge–discharge (GCD). EIS was performed at an amplitude of 5 mV and in the frequency range of 1–100,000 Hz. The electrochemical performances of CFASCs were evaluated using a two-electrode system. The area energy density (Wh cm^−2^) and area power density (W cm^−2^) were determined based on the area specific capacitance [32,33].

(1) $$E=\left(C_{S}∆V^{2}\right)/\left(2\times 3.6\right)$$

(2) P =3600E/∆t

During the leakage current measurement, the CFASCs were charged to 1.6 V and kept at this potential for 60 min. Once the 60 min period elapsed, the stabilized current flowing through the CFASCs offset the self-discharge current, allowing the measured value to be regarded as the leakage current.

## 3. Results

### 3.1. Structure and Morphology of the Fiber-Shaped PANI/Graphene@CNT Electrode

**Figure 1:**
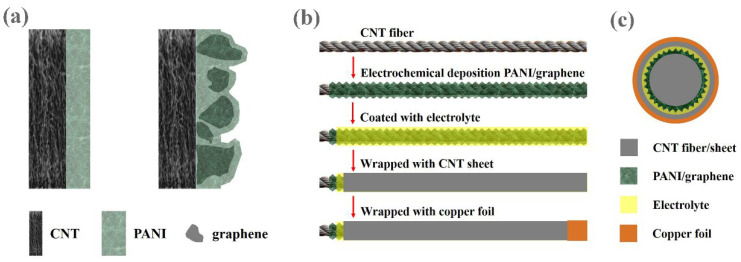
(**a**) Microstructure image of PANI@CNT and PANI/graphene@CNT samples; (**b**) illustration of the manufacturing steps for the ACFSCs; (**c**) the cross-sectional structure of the CFASCs.

Generally, conductive polymer polyaniline (PANI) films grown through electrochemical polymerization are dense and brittle. Nevertheless, after the incorporation of micron-sized graphene sheets, the graphene sheets will migrate toward the substrate during the electrochemical deposition process, while the conductive polymer PANI simultaneously polymerizes and grows on both the conductive substrate and the surface of the graphene sheets. This dual growth mechanism leads to an uneven surface morphology, characterized by numerous micropores extending from the PANI surface into its interior, as illustrated in Figure 1a.

**Figure 2:**
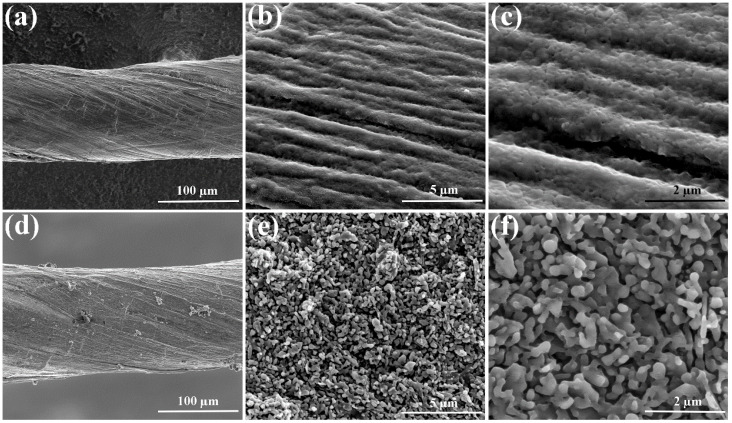
SEM comparison images of PANI@CNT (**a**–**c**) and PANI/graphene@CNT (**d**–**f**) electrode materials under different magnification.

Figure 2 shows the SEM comparison images of PANI@CNT and PANI/graphene@CNT electrode materials. As shown in Figure 2a–c, the PANI layer is uniformly and densely coated on the surface of the CNT fiber, and the PANI@CNT composite largely retains the original microstructure of the CNT fiber surface. In contrast, numerous lamellar microstructures are distributed across the surface of the PANI/graphene@CNT composite electrode (Figure 2d–f). Additionally, there are micropores of varying sizes among the lamellar microstructures, which is consistent with previous predictions. The highly porous structure of PANI/graphene provides multiple functional advantages: (1) It substantially increases the specific surface area of PANI, thereby enhancing the interaction between the active material and the electrolyte; (2) The existence of micropores creates efficient channels for electrolyte infiltration, establishing favorable conditions for ion diffusion and migration within the polymer matrix; (3) It converts the originally dense and brittle PANI layer into a sponge-like morphology with improved mechanical flexibility; (4) The abundant void spaces within the composite can effectively accommodate volumetric changes in PANI during charge–discharge cycles, thus mitigating the degradation of the active PANI layer; (5) The incorporation of graphene significantly enhances the electrical conductivity of PANI, promoting efficient electron transport and reducing the electrode’s internal resistance.

**Figure 3:**
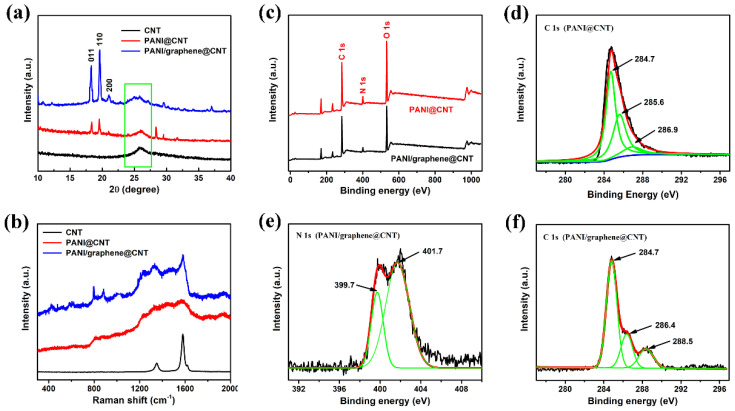
(**a**) XRD and (**b**) Raman spectra of CNT fiber, PANI@CNT and PANI/graphene@CNT samples XPS spectrogram analysis of PANI@CNT and PANI/graphene@CNT samples: (**c**) Full spectrum; (**d**) PANI@CNT C 1s of the sample; (**e**,**f**) PANI/graphene@CNT N 1s and C 1s of the sample.

Figure 3a presents the X-ray diffraction (XRD) patterns of the CNT fibers, PANI@CNT, and PANI/graphene@CNT samples. As observed, both the PANI@CNT and PANI/graphene@CNT samples exhibit three prominent diffraction peaks at 18.2°, 19.6°, and 21.0°, corresponding to the (011), (110), and (200) crystallographic planes of fully reduced PANI, respectively, thereby confirming the presence of PANI in the composites [34]. All three samples display a broad diffraction peak near 26°, which corresponds to the (002) surface of the carbon nanotube material [35,36]. Notably, the diffraction peak of the PANI/graphene@CNT sample exhibits a slight shift toward higher angles, which can be reasonably attributed to the incorporation of graphene within the composite matrix. Figure 3b presents the Raman spectra of the CNT fibers, PANI@CNT, and PANI/graphene@CNT samples. The Raman spectrum of CNT fibers shows two typical bands at 1350 and 1580 cm^−1^, corresponding to the D band (related to defects) and G band (related to graphite carbon) of carbon materials, respectively [37]. For the PANI@CNT and PANI/graphene@CNT samples, there is a series of characteristic peaks between 1100 cm^−1^ and 1600 cm^−1^, among which the peaks at 1161 cm^−1^, 1255 cm^−1^, 1346 cm^−1^, 1481 cm^−1^, and 1583 cm^−1^ are attributed to imine deformation, in-plane C–H bending, C–N+ stretching vibration, stretching vibration of the C=N quinone ring, and C=C stretching of the benzene ring, respectively [38]. Due to the presence of graphene, the characteristic peaks at 1350 cm^−1^ and 1580 cm^−1^ of the PANI/graphene@CNT sample are particularly prominent compared to that of PANI@CNT. Such results are consistent with XRD, jointly confirming the existence of graphene.

In addition, XPS was used to study the chemical states of the atoms contained in the PANI/graphene@CNT sample. Figure 3c shows the full XPS spectrum of the samples, where peaks of C 1s, N 1s, O 1s, S 2s, and S 2p can be detected. The presence of O and S elements is due to the residual sulfate ions during sample preparation. The C 1s peak of the PANI@CNT sample can be divided into three peaks through Gaussian–Lorentzian fitting (Figure 3d), with binding energies of 284.7 eV, 285.6 eV, and 286.9 eV, corresponding to sp^2^ hybridized C atoms, C–N single bonds, and C=N double bonds, respectively [39]. For comparison, the PANI/graphene@CNT sample exhibits a new characteristic peak at approximately 288.5 eV (Figure 3f), which is attributed to the incorporation of the C=O bond from the carbonyl group during the functionalization of graphene. Similarly, the N 1s peak can be divided into two peaks t (Figure 3e), with a binding energy of 399.9 eV corresponding to the N atoms in the amine of the benzene ring (–NH–) and a binding energy of 401.3 eV corresponding to the positively charged N atoms (–NH^+^=) [40,41].

### 3.2. Electrochemical Performance of the Fibrous PANI/Graphene@CNT Samples

**Figure 4:**
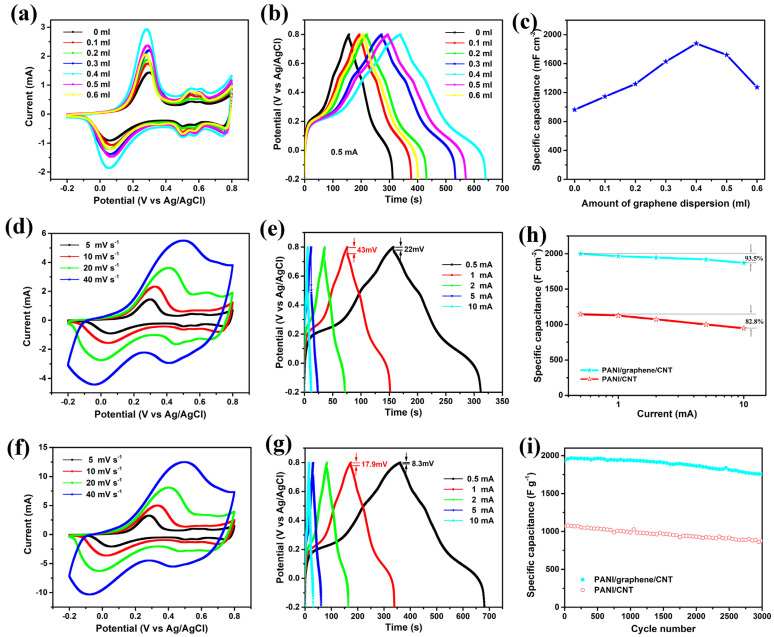
The electrochemical properties of the PANI/graphene@CNT composite electrode fabricated with different graphene content are analyzed as follows: (**a**) CV curve with a scanning rate of 5 mV s^−1^; (**b**) GCD curve at 0.5 mA; (**c**) the effect of varying graphene content on the areal specific capacitance of the sample; (**d**,**e**) CV curves and GCD curves of PANI@CNT composite electrode; (**f**,**g**) CV curves and GCD curves of PANI/graphene@CNT composite electrode were obtained by adding 0.4 mL graphene dispersion solution; (**h**) The area specific capacitance at different current densities, and (**i**) the cycling performance of the PANI@CNT and PANI/graphene@CNT composite materials.

The introduction of graphene has a significant impact on the electrochemical performance of the PANI shell. On the one hand, the addition of graphene alters the surface morphology of the PANI shell, thereby increasing the loading of active materials and the specific surface area. On the other hand, the introduction of graphene improves the conductivity of the PANI shell. To investigate the effect of graphene content on the electrochemical performance of the PANI/graphene@CNT composite electrode, we regulated and optimized the electrochemical performance of the composite electrode by adding different amounts of graphene dispersion to the precursor solution. Figure 4a shows the cyclic voltammetric (CV) curves of PANI/graphene@CNT composite electrodes prepared by adding different contents of graphene dispersion at a scan rate of 5 mV s^−1^, and a voltage window of −0.2 to 0.8 V. The area enclosed by the CV curves first increases and then decreases with the increase in graphene content, reaching the maximum when the graphene addition is 0.4 mL. This suggests that the content of graphene plays a crucial role in determining the electrochemical properties of the electrode material. In addition, a pair of obvious redox peaks and two pairs of weak redox peaks can be observed from the CV curves, indicating its typical pseudocapacitive characteristics. PANI undergoes conversion between the reduced state, semi-reduced states (mononitro, dinitro, and trinitro forms), and the oxidized state (tetranitro form), thereby generating capacitance related to the electrode charging and discharging potential. Figure 4b presents the GCD curves of the PANI/graphene@CNT composite electrodes with varying graphene contents at a current of 0.5 mA and a voltage window of −0.2 to 0.8 V. As observed, the discharge time initially increases and subsequently decreases with increasing graphene content, reaching a maximum when 0.4 mL of graphene is incorporated. This trend is fully consistent with the results obtained from the CV measurements. Moreover, the plateau potential observed in the GCD curves aligns with the peak voltage of the redox peaks in the CV curves, further confirming the pseudocapacitive behavior of the electrode material.

According to the GCD curves of PANI/graphene@CNT composite electrode with different graphene content, the area specific capacitance is further calculated using the following equation:

(3) $$C_{S}\;=\;\frac{I∆t}{S∆V}$$

As shown in Figure 4c, when the added amount of graphene increases from 0 mL to 0.6 mL, the specific capacitance values of the PANI/graphene@CNT composite electrodes are 962.5 µF cm^−2^, 1144.4 µF cm^−2^, 1318.8 µF cm^−2^, 1627.5 µF cm^−2^, 1878.1 µF cm^−2^, 1720 µF cm^−2^, and 1273.1 µF cm^−2^, respectively. When the amount of added graphene increased from 0 mL to 0.4 mL, the area-specific capacitance of the prepared composite electrode samples increased approximately linearly. On the one hand, the incorporation of graphene alters the surface morphology of the PANI shell, thereby enhancing the loading capacity and specific surface area of the active material. On the other hand, graphene improves the electrical conductivity of the PANI shell, which facilitates the surface redox reactions. However, when the graphene content was further increased from 0.4 mL to 0.6 mL, the area-specific capacitance began to decrease significantly. This decline is attributed to the excessive accumulation of graphene microstructures on the electrode surface, which hinders ion diffusion in the electrolyte and increases the diffusion resistance.

To further elucidate the effect of graphene incorporation on the electrochemical performance of the composite electrode, CV and GCD tests were conducted on the PANI@CNT and PANI/graphene@CNT samples, respectively. A comparison of the CV curves at scan rates ranging from 5 to 40 mV s^−1^ (Figure 4d,f) indicates that both electrode materials display similar curve shapes with a pair of well-defined redox peaks, but the PANI/graphene@CNT sample exhibits a larger integrated area. As the scan rate increases, the overall shape of the CV curves remains largely unchanged, indicating good rate capability of the prepared composite electrode. Additionally, a slight shift in the redox peak positions is noticed as the scan rate increases, which can be attributed to polarization effects arising from the internal resistance of the material. The two pairs of weaker redox peaks that appear at lower scan rates gradually vanish at higher scan rates. This phenomenon is likely due to the excessively high scan rate, which limits the occurrence of less active redox reactions in a timely manner.

Figure 4e,g, respectively, present the constant–current charge–discharge curves of the PANI@CNT and PANIgraphene@CNT composite electrode materials at various current densities. These results indicate that the incorporation of graphene significantly prolongs the discharge time of the composite electrode. Moreover, the platform voltage potential observed in the GCD curves aligns with the positions of the redox peaks in the CV curves. To provide a more intuitive illustration of the effect of graphene on the electrochemical performance of PANI-based materials, the area-specific capacitances of the PANI@CNT and PANIgraphene@CNT composite electrodes at different current densities were calculated (Figure 4h). As the current density increases from 0.5 mA to 10 mA, the specific capacitance of the PANI@CNT composite retains 82.8% of its initial value, whereas that of the PANIgraphene@CNT composite retains 93.5% of its initial value. This clearly demonstrates that the incorporation of graphene enhances the rate capability of the composite electrode. This improvement can be primarily attributed to the reduced internal resistance of the PANI material upon graphene integration, which facilitates electron transport within the composite and promotes the redox reactions occurring on the PANI surface, thus improving the rate performance of the hybrid electrode. The cycle life of electrode materials is closely related to the practical application potential of supercapacitor devices. Figure 4i presents the cycling stability performance of the PANI@CNT and PANIgraphene@CNT composite electrodes, were performed at a current density of 2 mA. The PANI@CNT and PANIgraphene@CNT composite materials retain 82.04% and 90.84% of their initial capacitance after 3000 cycles, respectively. This demonstrates that the incorporation of graphene significantly enhances the cycling stability of the electrode material. The improved cycling stability of the PANI/graphene@CNT electrode can be attributed to its superior electrical conductivity, which facilitates uniform charge distribution during the charge–discharge process and prevents microstructural degradation caused by local overpolarization.

### 3.3. Electrochemical Evaluation of the CFASC Devices

**Figure 5:**
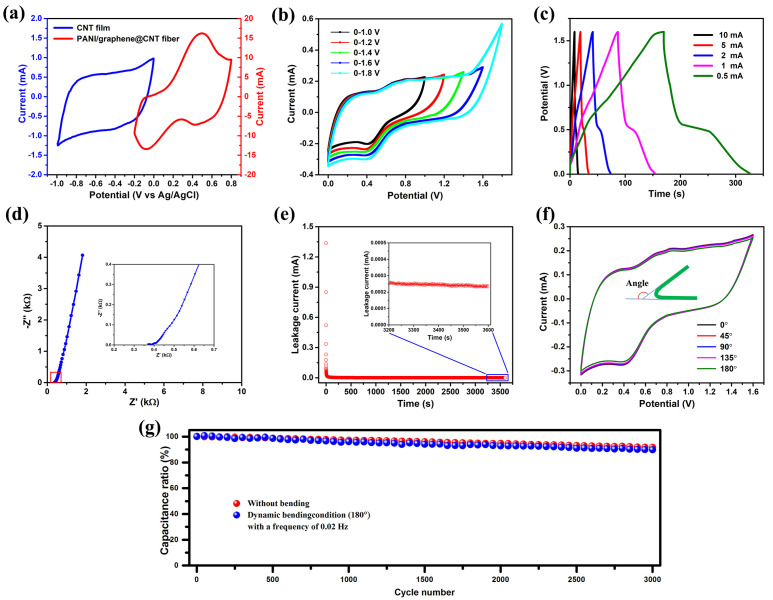
(**a**) CV curves of fabricated hybrid electrode and CNT-films at 50 mV s^−1^ and their corresponding voltage Windows; (**b**) CV curves of PANI/graphene@CNT//CNT-film CFASC under various voltage windows at 20 mV s^−1^; (**c**) GCD curves of the CFASC devices under various current from 0.5 mA to 10 mA; (**d**) Nyquist spectrum of CFASC device; (**e**) leakage current test curve of CFASC device, the inset is the enlarged view of the content in the red square; (**f**) CV curves of the device under different degrees of bending at a scanning rate of 20 mV s^−1^, and the inset is the schematic diagram of the bending angle of the devices; (**g**) mechanical stability test results of the device after 3000 bends.

To further evaluate the performance of the PANI/graphene@CNT composite electrode in practical applications, a coaxial fiber-shaped asymmetric supercapacitor (CFASC) was successfully fabricated using PANI/graphene@CNT fiber, CNT film, and H_2_SO_4_/PVA gel used as the cathode, anode, and electrolyte, respectively. As shown in Figure 5a, the PANI/graphene@CNT and CNT films exhibit distinct voltage windows, which are (−0.2 to 0.8 V) and (−1.0 to 0.0 V), respectively. This suggests that the two materials can be assembled into an asymmetric supercapacitor configuration, potentially increasing the device’s operating voltage to 1.8 V. Figure 5b presents the CV curves of the fabricated CFASC device at different voltage windows at a scan rate of 20 mV s^−1^. As expected, the voltage window of the device can be extended up to 0–1.8 V. However, under the 0–1.8 V voltage window, the CV curve exhibits significant distortion when the voltage window enlarges to 1.8 V, indicating that the electrode material undergoes notable polarization at high voltage. Such polarization phenomena suggest the occurrence of irreversible chemical reactions on the surface of the electrode material, which is unfavorable to the stability of the device. The specific capacity of the CFASCs device can be determined based on the galvanostatic charge-discharge (GCD) curve of the device (Figure 5c), from which the energy density and power density of the CFASCs device can be further derived through Equations (1) and (2). The maximum energy density and power density could reach 160.5 µWh cm^−2^ and 13 mW cm^−2^, respectively, which is considerably higher than those of previously reported FSCs (Table 1), proving its excellent energy storage and output capabilities. Figure 5d presents the Nernst plot of the fabricated CFASC device, showing an equivalent series resistance (ESR) of 362 Ω, which is significantly higher than that of traditional planar supercapacitors. However, compared to previously reported fiber-shaped supercapacitors, the ESR of our device is notably lower, indicating a substantial improvement in performance. The relatively high ESR observed in fiber-shaped supercapacitors can be attributed to their compact size and the inherently higher resistance of fibrous electrodes.

The self-discharge phenomenon caused by leakage current is a critical factor affecting the energy storage performance of supercapacitors, which significantly compromises the lifespan and reliability of the devices. The occurrence of leakage current results from the combined effects of the electrolyte’s internal resistance, impurities in the electrode materials, and interfacial impedance. As shown in Figure 5e, the current decreases rapidly during the initial stage and then stabilizes. The current magnitude after 1 h is 0.25 µA, representing the leakage current of the CFASCs. Compared to the milliampere-level leakage currents typically observed in conventional planar supercapacitors, the leakage current in our fabricated devices is reduced by 4 orders of magnitude, which is expected to greatly enhance the service life and reliability of the devices.

The mechanical and electrochemical stability of the CFASC device was evaluated using an integrated system consisting of a custom-built linear motor and an electrochemical workstation. The test was divided into static bending and dynamic bending modes. The fundamental principle of the test is as follows: the fabricated CFASC device was fixed onto the linear motor and bent to various angles, either once (static bending) or repeatedly (dynamic bending). Simultaneously, the electrochemical workstation was employed to monitor the device’s performance changes in real time, thereby assessing its mechanical and electrochemical stability. In the static bending tests, the device was bent from 0° to 180°, and the CV curves remained nearly identical at 20 mV s^−1^ under various bending conditions (Figure 5f). The measured capacitance change remained below 1% across the entire bending range, indicating consistent electrochemical performance. Furthermore, the durability of the CFASC device under repeated bending cycles was investigated (Figure 5g), with GCD measurements recorded every 50 bending cycles at a current density of 50 μA. With an increasing number of bending cycles, a steady decrease in capacitance was observed. Nevertheless, the device retained 89.77% of its initial capacitance after 3000 bending cycles, and no structural damage was observed. The findings demonstrate that the hybrid electrode exhibits excellent mechanical and electrochemical stability under both static and dynamic bending conditions. This performance can be attributed to the intrinsic flexibility and the thoughtfully engineered design of the PANI/graphene@CNT fiber-based electrode.

## 4. Conclusions

In this study, porous PANI/graphene composite films were successfully deposited onto the surface of CNT fibers through an electrochemical polymerization method, and the specific capacitance of the composite electrode reaches 1878.1 µF cm^−2^ at a current density of 0.5 mA. The incorporation of graphene significantly enhances the electrochemical performance of the composite electrode. The high specific surface area and hierarchical porous structure not only increase the contact area between the electrolyte and the active materials but also facilitate the rapid diffusion and transport of ions. Based on the different voltage windows of the positive and negative electrode materials, a coaxial fiber-shaped asymmetric supercapacitor (CFASC) was fabricated, and its voltage window was extended to 1.8 V. The maximum energy density and power density could reach 160.5 µWh cm^−2^ and 13 mW cm^−2^, respectively, proving its excellent energy storage and output capabilities. More importantly, the prepared CFASC device showed remarkable mechanical and electrochemical durability. Even after 3000 bending cycles, it retained 89.77% of its original capacitance, highlighting its promising applicability in the realm of flexible electronics. In summary, this study successfully developed a high-performance fibrous asymmetric supercapacitor through material design and device optimization, providing new ideas and technical support for the development of flexible energy storage devices.

## Figures and Tables

**Table 1:** Electrochemical performances of recently reported fiber-shaped SCs.

Electrode Material	Electrolyte	Voltage Window	$E_{i}$	$P_{i}$	Ref.
PANI/graphene@CNT//CNT	PVA/H_2_SO_4_	0–1.6 V	160.5 µWh cm^−2^	13.0 mW cm^−2^	This work
MnO2@ZnO@CNT//CNT	PVA/H_2_SO_4_	0–1.6 V	20.7 µWh cm^−2^	0.329 mW cm^−2^	[4]
N-doped RGO-SWCNT composite fiber	PVA/H_3_PO_4_	0–1 V	16.1 µWh cm^−2^	2.84 mW cm^−2^	[6]
MWCNT@CMF//CNT film	PVA/H_3_PO_4_	0–1 V	9.8µW h cm^−2^	189.4 µW cm^−2^	[8]
RGO-fiber//RGO-fiber	PVA/H_2_SO_4_	0–0.8 V	0.17 µWh cm^−2^	0.1 mW cm^−2^	[10]
CNT//CNT	PVA/H_2_SO_4_	0–0.8 V	0.226 µWh cm^−2^	0.493 mW cm^−2^	[12]
OMC-MWCNT composite fiber	PVA/H_3_PO_4_	0–1 V	1.77 µWh cm^−2^	0.032 mW cm^−2^	[15]
SWCNT-AC composite fiber	PVA/H_2_SO_4_	0–0.8 V	3.29 µWh cm^−2^	3.36 mW cm^−2^	[16]

## Data Availability

The original contributions presented in this study are included in the article. Further inquiries can be directed to the corresponding author(s).
